# Effects of Foraging and Provisioning Behavior on Offspring Development in the Ground Nesting Carpenter Bee *Xylocopa* (*Proxylocopa*) *mongolicus* (Hymenoptera, Apidae, Xylocopini)

**DOI:** 10.3390/insects17040388

**Published:** 2026-04-02

**Authors:** Xuan Liu, Chunling He, Dongshuo Yang, Le Yang, Jiabao Wei, Qianlei Dai, Jia Wan, Jialin Li, Yaheng Ma, Kaiyue Zhang

**Affiliations:** 1 College of Horticulture and Plant Protection, Henan University of Science & Technology, Luoyang 471000, China; 15036679391@163.com (X.L.); yl1162568764@163.com (L.Y.); 13592082539@163.com (J.W.); d1713821911@163.com (Q.D.); 18438669750@163.com (J.W.); jialinli2026@163.com (J.L.); mayahengabc@163.com (Y.M.); zhangkaiyue95@126.com (K.Z.); 2 College of Life Sciences, Luoyang Normal University, Luoyang 471934, China; yangdongshuo@126.com

**Keywords:** *Proxylocopa*, floral resources, bee bread, desert environment, *Ammopiptanthus mongolicus*

## Abstract

Understanding how pollinators adjust their behavior to maximize reproductive success in resource-limited desert ecosystems is a fundamental ecological question. In this study, we investigated *X. mongolicus* using a combination of field behavioral observations, pollen identification, nutritional analysis, and morphometric measurements to systematically examine interannual variations in its flower visitation spectrum, foraging behavior, bee bread composition, and offspring body size. Our findings reveal a striking contrast: although this species exhibits polyphagy in flower visitation, it shows dietary specialization in larval nutrition—over 92% of the pollen in its bee bread originates from just two leguminous species, *A. mongolicus* and *O. bicolor*. Foraging duration increased with rising temperature and humidity, and bees adapted to strong winds by flying close to the ground. Compared with 2024, the bee bread in 2025 had lower fresh weight but higher crude protein content, and offspring body size was larger—likely due to more abundant spring rainfall in 2025, which improved the flowering performance of *A. mongolicus*. Collectively, these results indicate that this bee species copes with resource uncertainty in desert habitats through flexible foraging range and highly specialized food storage, with its reproductive success closely tied to the nutritional supply of key plants and precipitation patterns. This study highlights the role of precipitation timing in shaping the nutritional foundation of plant–pollinator interactions, providing a scientific basis for the conservation and management of desert pollinators.

## Simple Summary

*Xylocopa mongolicus* is an important pollinator in desert ecosystems. It employs a composite strategy of generalist foraging with narrow brood-provisioning specialization to adapt to desert environments. The species visits flowering plants from 10 families and 17 species, yet the pollen in its bee bread is highly concentrated in *Ammopiptanthus mongolicus* and *Oxytropis bicolor* (together accounting for >92%). Foraging duration is significantly regulated by temperature and humidity and varies markedly between years with contrasting precipitation. In 2025, which experienced more abundant spring rainfall, the bee bread was lighter in weight but higher in protein content, and offspring body size was significantly larger than that in 2024. These findings indicate that the reproductive success of this species is closely linked to the nutritional supply of key plants and precipitation patterns, offering important insights into the adaptation mechanisms of desert pollinators to climatic fluctuations.

## 1. Introduction

All animals require food. In environments with limited and variable resources, animals have evolved foraging strategies to obtain nutrients in amounts and balances that maximize fitness traits [1], such as body size [2,3,4], development [5], reproduction [6,7,8], and sex ratio [9]. In desert ecosystems, pollinators confront a suite of harsh conditions such as drastic temperature fluctuations, scarce precipitation, frequent strong winds, and consequently, low productivity and high spatiotemporal heterogeneity in plant resources [10,11,12,13,14,15]. Collectively, these pressures rigorously test pollinator foraging efficiency and energy balance [16,17].

Wild bees constitute a group of highly protected and essential pollinators [18,19,20,21]. Their relatively short life cycles and limited spatiotemporal ranges for habitation, nesting, and foraging make them exceptionally sensitive to environmental changes [7,19,22,23,24,25,26,27]. Their reproductive success fundamentally hinges on the resource utilization strategies they adopt. A key aspect is adult foraging behavior, which directly dictates the quality of food provisions for their offspring [28], thereby profoundly influencing larval developmental health and population fitness [29].

The genus *Xylocopa* (large carpenter bees), exhibiting social polymorphism that ranges from solitary to primitively eusocial, has attracted considerable attention in the fields of pollination ecology and adaptive evolution [30,31,32,33,34]. Widely distributed across diverse global habitats, many species within this group show significant adaptations to arid and semi-arid environments [11,13,35,36]. Research indicates that the morphological and functional traits of carpenter bees (e.g., body size and heat tolerance) are closely correlated with their floral visitation choices and pollination efficiency, often making them key or even dominant pollinators in desert ecosystems [11,37]. Their strong flight capability and broad foraging range facilitate the maintenance of pollination services in habitats with severely fragmented resources [38]. In recent years, studies on the foraging strategies, reproductive investment, and habitat selection of *Xylocopa* have provided crucial insights into the survival and adaptation mechanisms of large-bodied bees in fragile ecosystems [17,35,39].

The subgenus *Proxylocopa* is the only group within the genus *Xylocopa* that nests in soil [32,35]. It occurs in desert regions from Albania, Greece, and Israel eastward to western China [32]. Of the 16 described species worldwide [32], ten occur in the desert regions of northwest China, including Inner Mongolia, Gansu, Xinjiang, and Ningxia [40], making them important solitary pollinators in arid ecosystems [16,37]. Females independently undertake all tasks of nesting, foraging, and brood care, establishing them as an ideal model for investigating the trade-offs between foraging and reproductive investment at the individual level [26,35,40]. Desert and semidesert environments impose a rather narrow window of activity because of the large temperature differences between day and night. *Xylocopa olivieri*, a ground-nesting species in semi-desert habitats, exhibits a distinctly bimodal daily foraging pattern under the semidesert climate conditions of Israel, foraging intensively in the early morning and evening but ceasing activity during midday [16]. *Xylocopa* (*Proxylocopa*) *mongolicus* is a dominant bee species in the desert steppes of northern Asia. However, its detailed foraging ecology, particularly how it adaptively forages under harsh environmental conditions, remains poorly understood.

This study focuses on the ground-nesting carpenter bee *X. mongolicus* and aims to address the following key questions: (1) What is the pollen spectrum of foraging and provisioning in *X. mongolicus*? (2) How do micro-environmental factors such as temperature, humidity, and wind speed affect its provisioning behavior? (3) How do the composition and nutritional quality of bee bread influence offspring development? Through behavioral observations and nutritional analyses, we seek to elucidate how this desert bee copes with environmental stress via behavioral and physiological adaptations and to provide new empirical evidence for the conservation of wild bee resources.

## 2. Materials and Methods

### 2.1. Study Area and Habitats

**Figure 1:**
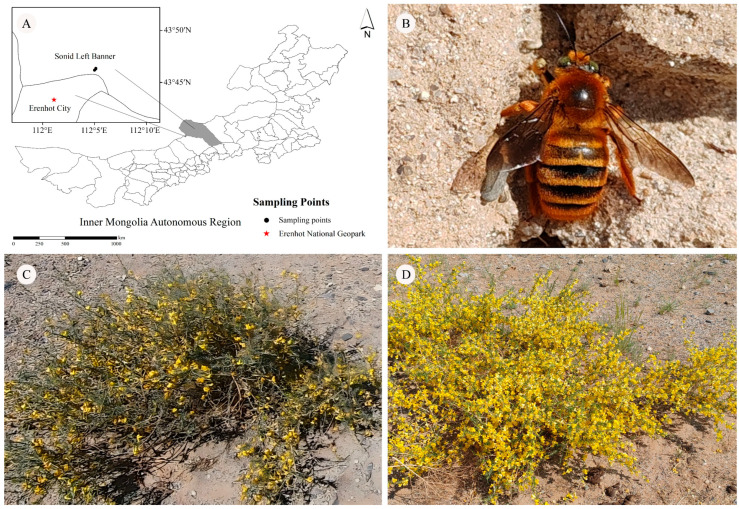
Overview of the Study Area. (**A**) Sampling site; (**B**) females of *Xylocopa mongolicus* at nesting sites; flowering status of *Ammopiptanthus mongolicus* on 19 May 2024 (**C**) and 25 May 2025 (**D**).

The study was conducted at the Erlian Cretaceous Dinosaur National Geopark in Erenhot City, Inner Mongolia Autonomous Region, located within the Erlian Basin of the arid desert region in northwest China (43°46′22″ N, 112°5′7″ E, elevation approximately 890 m) (Figure 1A). The geopark covers a total area of 10,471 hectares, with a core zone of 29.9 hectares (Figure 1B). The regional climate is characterized by a temperate continental monsoon and arid desert steppe climate, featuring perennial aridity, low rainfall, and frequent winds. The number of days with wind speeds ≥ grade 8 (Beaufort scale) ranges from 71.8 to 85.5 per year, with wind intensity being particularly pronounced in spring. The mean annual precipitation is only 142 mm, while evaporation reaches 2695 mm, approximately 17 times the precipitation. The average annual wind speed is about 17 m/s. Precipitation data from the National Meteorological Information Center indicate that snowfall in the winters of 2023 and 2024 was 12.9 mm and 2.9 mm, respectively; rainfall in the springs of 2024 and 2025 was 7.8 mm and 15.7 mm, respectively (https://data.cma.cn).

The dominant soil type is light brown calcareous soil mixed with sandy gravel, with scattered depressions locally that may accumulate temporary water during the rainy season. The vegetation in the study plots is sparse, dominated by *A. mongolicus* shrubland, and accompanied by scattered flowering plants [15] (Figure 1C,D).

### 2.2. Floral Species Survey and Identification

From mid-July 2023, mid-May 2024, as well as mid-May and late July 2025, all plant species visited by the *X. mongolicus* were recorded within a 3 km radius of their nesting sites using the direct focal-animal observation method (unpublished data: The peak provisioning period for female *X. mongolicus* occurs in middle to late May, while newly emerged adults require critical nutrition supplementation in early to middle July) (Figure 1B). Concurrently, flower bud samples from all flowering plants within the sampling area were collected, placed in clean paper bags, and brought back to the laboratory for subsequent use. Based on corolla diameter, the visited flowers were categorized into three types: small (<1 cm), medium (1–4 cm), and large (>4 cm) [11,41]. Plant species identification was conducted with reference to *Flora of China*, *Flora of Inner Mongolia*, and online databases.

### 2.3. Observation of Foraging Behavior

On sunny days (19–21 May 2024 and 26 May 2025), the foraging behavior of female bees was recorded using visual observation and video recording. A stopwatch was used to time the duration of foraging trips (for collecting a pollen-nectar mixture) and the time spent unloading this mixture inside the nest [23]. Based on the daily activity pattern of *X. olivieri*, a ground-nesting species of the subgenus *Proxylocopa* in semi-desert environments, observations were conducted during two daily time windows: morning (8:00–13:00) and afternoon (16:00–19:00) [16,17]. Each day, three female bees were observed at the same site. Concurrently, temperature, humidity, and wind speed were measured with a handheld weather meter (Kestrel 3500, NK, Boothwyn, PA, USA). Foraging trip duration was defined as the time required for an individual female bee to depart from the nest and return carrying pollen and nectar [42]. Only the foraging behavior of females with pollen visible on their hind legs was considered. Data from all observation days were pooled to analyze the foraging behavior of the carpenter bees and to assess the influence of climatic factors on this behavior.

### 2.4. Bee Bread Sample Collection and Composition Analysis

Samples of bee bread were collected at the nesting sites of *X. mongolicus* from 19 to 22 May 2024 and from 21 to 23 May 2025. The reproductive nests were carefully opened using a scalpel, and the offspring cells from each nest were placed into a collection box and transported to the laboratory [35]. In the laboratory, each cell was carefully dissected, and the fresh bee bread corresponding to offspring at the egg stage was weighed as a sample representing the bee bread weight consumed per offspring. Twenty such samples were collected each year. Samples for compositional analysis were obtained from uneaten bee bread left after larval mortality during rearing. This bee bread was transferred into clean 1.5 mL cryotubes and stored at −20 °C for later use.

Pollen types in bee bread were identified, and their relative abundances were statistically analyzed by comparing pollen morphology with that of flowering plants from the sample plots, using both light microscopy and scanning electron microscopy (FlexSEM 1000, Hitachi, Ltd., Tokyo, Japan) [43,44]. The specific methodology was as follows. An appropriate amount of bee bread sample was weighed into a 2 mL centrifuge tube. Subsequently, 1.5 mL of sterile water was added, and the mixture was dissolved in a 40 °C water bath for 5 min, followed by vortex mixing for 5 min to achieve complete homogenization. From the resulting suspension, 1 µL was taken and dropped onto a glass slide; three such slides were prepared for each sample. Under an optical microscope (Leica DM 2500, Leica Microsystems, Wetzlar, Germany), five fields of view were randomly selected per slide (each containing no fewer than 100 pollen grains) for counting. The counts from the five fields of view were first pooled to obtain the total count for each individual slide. The relative abundance—defined as the percentage of a specific pollen type relative to the total number of pollen grains—was then calculated independently for each slide. Finally, these values were averaged across the three replicate slides to determine the final relative abundance for each sample.

The contents of carbon (C) and nitrogen (N) in bee bread were determined using an elemental analyzer (FlashSmart CHNS/O, Thermo Fisher Scientific Inc., Waltham, MA, USA). Subsequently, the crude protein content and the carbon-to-nitrogen ratio (C/N) were calculated. The specific method was as follows: 1–3 mg of bee bread sample, weighed on an electronic balance (XPR2U/AC, Mettler-Toledo International Inc., Columbus, OH, USA, precision: 0.001 mg), was placed into a dedicated tin capsule. After recording the mass, air was expelled, and the capsule was folded into a compact ball and loaded into the autosampler. Analysis was performed with the combustion tube set at 1060 °C, the reduction tube at 950 °C, a helium flow rate of 140 mL/min, and an oxygen flow rate of 250 mL/min. Crude protein content was calculated as 6.25 times the nitrogen content, where 6.25 represents the conventional protein conversion factor [45,46]. The C/N ratio was defined as the total carbon mass divided by the total nitrogen mass.

### 2.5. Rearing of Offspring and Measurement of Adult Morphological Traits

The offspring nesting cells collected in 2024 and 2025 were placed in rearing containers (bottom diameter: 3 cm, lid diameter: 4 cm, height: 3 cm) and maintained in an incubator at 28 °C and 45% relative humidity until adult emergence. Upon emergence, adults were transferred to a −20 °C freezer for 2 h to ensure complete mortality, after which they were retrieved for measurement. A digital caliper (DL91150, Deli Group Co., Ltd., Ningbo, China, precision: 0.01 mm) was used to measure the intertegular distance (ITD) [47] and head width [48] of the adults.

### 2.6. Data Analysis

All data were compiled and statistically organized using Excel 2019. Statistical analyses were performed with SPSS 20.0 (version 20.0). Prior to analysis, all variables were tested for normality using the Shapiro–Wilk test and for homogeneity of variances using Levene’s test. The Mann–Whitney *U* test was employed to analyze significant differences in bee bread weight between different years because these data did not meet the assumption of normality. Independent samples *t*-tests were used to examine significant differences between years for the following variables, which met the required parametric assumptions: foraging trip duration (time spent collecting pollen-nectar), unloading time, crude protein content in bee bread, and the carbon-to-nitrogen (C/N) ratio. Least squares linear regression analysis was conducted with foraging trip duration as the dependent variable and temperature, humidity, and wind speed as independent variables. Figures were generated using Origin 2022 and GraphPad Prism 10.1.2. Data are presented as mean ± standard deviation, and the significance level was set at α = 0.05.

## 3. Results

### 3.1. Foraged Plant Species

**Figure 2:**
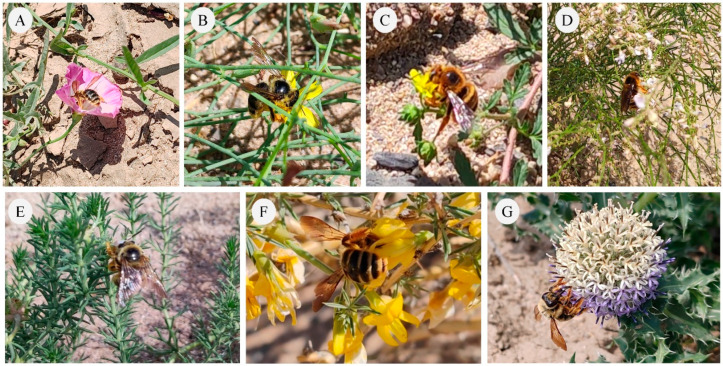
Foraging plant species of *X. mongolicus* in Erenhot, Inner Mongolia, China. (**A**) Visiting *Convolvulus arvensis*; (**B**) visiting *Lipschitzia divaricate*; (**C**) visiting *Potentilla supina*; (**D**) visiting *Astragalus melilotoides*; (**E**) visiting *Peganum harmala*; (**F**) visiting *Ammopiptanthus mongolicus;* (**G**) visiting *Echinops gmelinii*.

The *X. mongolicus* exhibited a broad foraging spectrum, visiting a total of 17 plant species belonging to 10 families. Among these, the families Fabaceae, Asteraceae, and Rosaceae were the most represented, each with 3 species. In terms of life form, herbaceous plants constituted the majority (82.35%). *A. mongolicus* was the only wild evergreen shrub recorded. Regarding floral traits, the most common characteristics were yellow corolla (35.29%), medium-sized flowers (41.18%), and actinomorphic symmetry (76.47%) (Table 1; Figure 2). These findings confirm its generalist ability in exploring and utilizing floral resources in desert ecosystems.

### 3.2. Characteristics of Provisioning Behavior and Their Environmental Response

**Figure 3:**
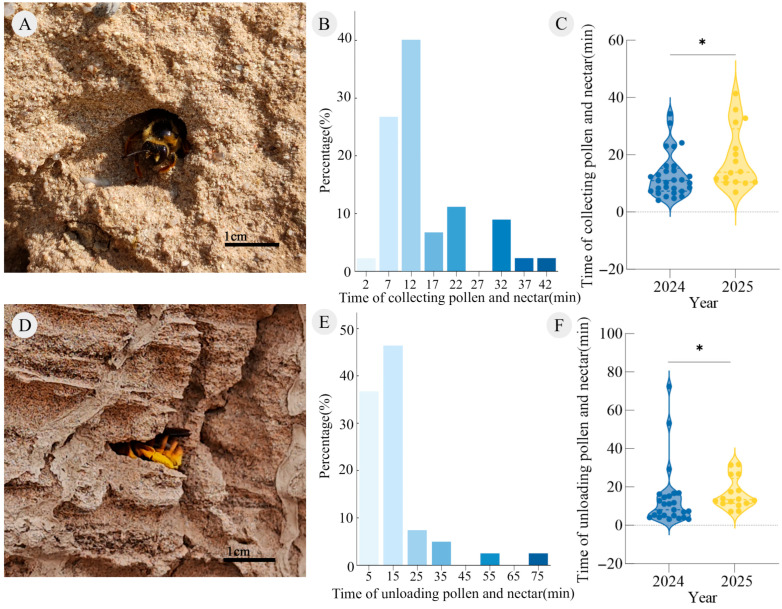
The provisioning behavior of *X. mongolicus*. (**A**) A female bee about to leave the nest; (**B**) frequency diagram of single foraging trips for pollen and nectar; (**C**) comparison of foraging times across different years; (**D**) a female bee carrying pollen back to her nest; (**E**) frequency diagram of the time female bees spend unloading pollen and nectar within the nest; (**F**) comparison of the female bees unloading pollen and nectar across different years. * *p* < 0.05.

The foraging trip duration of *X. mongolicus* ranged from 4.10 to 41.35 min (*n* = 45), with an average of 14.89 ± 9.25 min. The highest frequency (66.67%) occurred within the 4.5–14.5 min interval (Figure 3A,B). The unloading pollen-nectar duration in the nest ranged from 3.12 to 72.45 min (*n* = 41), with an average of 14.96 ± 13.55 min. The highest frequency (82.93%) fell within the 3.12–20 min interval (Figure 3D,E). There is a significant difference in the foraging trip duration of *X. mongolicus* between 2024 (range: 4.10–34.32 min, mean ± SD: 12.73 ± 7.67 min, *n* = 28) and 2025 (range: 6.93–41.35 min, mean ± SD: 18.80 ± 10.77 min, *n* = 17; independent samples *t*-test, t = −2.197, *p* = 0.033, Figure 3C). In contrast, no significant difference was detected in unloading pollen-nectar duration between 2024 (*n* = 25) and 2025 (*n* = 16). (independent samples *t*-test, t = −0.609, *p* = 0.546, Figure 3F).

**Figure 4:**
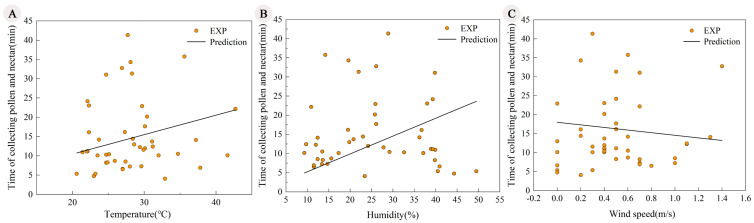
Effects of environmental factors on the foraging behavior of *X. mongolicus*. (**A**) Temperature; (**B**) humidity; (**C**) wind speed.

The nesting female bees of *X. mongolicus* first left the nest at 08:30 in the morning, with a minimum temperature of 17.4 °C, humidity of 11.2%, and wind speed of 0 m/s. The latest return to the nest occurred at 18:27 in the afternoon, with a minimum temperature of 19.1 °C, humidity of 10.6%, and wind speed of 0 m/s. The foraging trip duration of *X. mongolicus* showed significant positive correlations with both temperature (R^2^ = 0.72, *p* < 0.001) and humidity (R^2^ = 0.61, *p* < 0.001; Figure 4A,B), indicating that foraging trip duration increased under warmer and more humid conditions. In contrast, the foraging trip duration exhibited a decreasing trend with increasing wind speed, though no significant correlation was observed (R^2^ = 0.07, *p* = 0.16; Figure 4 C).

### 3.3. Pollen Types in Bee Bread

**Figure 5:**
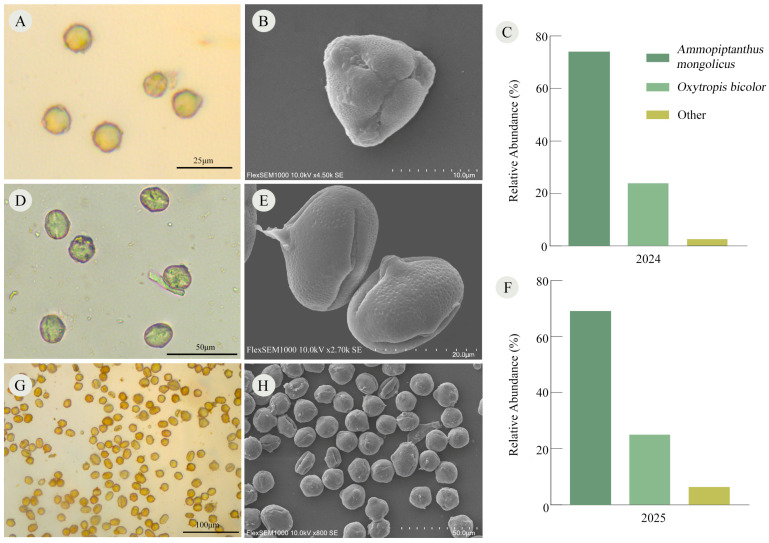
The microscopic morphology and species composition of the main pollen in the bee bread of *X. mongolicus*. (**A**,**B**) Microscopic morphology of *A. mongolicus* pollen; (**C**) 2024 pollen species composition in bee bread; (**D**,**E**) microscopic morphology of *Oxytropis bicolor* pollen; (**F**) 2025 pollen species composition in bee bread; (**G**,**H**) microscopic morphology of pollen in bee bread.

Pollen from six plant species was identified in the bee bread of *X. mongolicus*, yet its proportions are highly concentrated. Among these, pollen from *A. mongolicus* constituted the primary source, accounting for 73.85 ± 12.81% (*n* = 20) in 2024 and 68.94 ± 6.02% (*n* = 20) in 2025. *Oxytropis bicolor* served as the secondary source, contributing 23.65 ± 12.55% (*n* = 20) in 2024 and 24.75 ± 5.75% (*n* = 20) in 2025. Pollen from these two plant species collectively comprised over 92% of the total pollen in the bee bread, while the remaining plants together accounted for less than 8% (Figure 5).

### 3.4. Bee Bread Characteristics and Offspring Development

**Figure 6:**
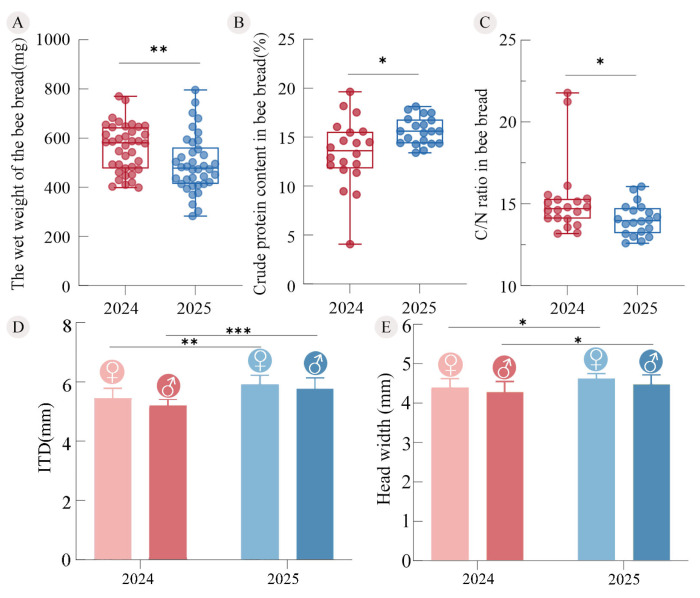
Bee bread wet weight and composition and offspring development in *X. mongolicus*. (**A**) Bee bread wet weight; (**B**) crude protein content in bee bread; (**C**) carbon-to-nitrogen ratio in bee bread; (**D**) intertegular distance (ITD) of female and male adults; (**E**) head width of female and male adults. ♀: female, ♂: male. * *p* < 0.05, ** *p* < 0.01, *** *p* < 0.001.

This climatic variation was further reflected in the characteristics of the bee bread in offspring nest cells of *X. mongolicus*. The fresh weight of bee bread in 2024 (range: 398.6–770.7 mg; *n* = 38) was significantly higher than that in 2025 (range: 282.5–796.3 mg; *n* = 38; Mann–Whitney *U* test, *Z* = −2.838, *p* = 0.005; Figure 6A). In contrast, the crude protein content of bee bread in 2025 (range: 13.40–18.13%; *n* = 20) was significantly higher than that in 2024 (range: 4.08–19.63%; *n* = 20; independent-samples *t*-test, t = −2.640, *p* = 0.014; Figure 6B). At the same time, the carbon-to-nitrogen ratio (C/N) of bee bread in 2025 (range: 12.60–16.05; *n* = 20) was significantly lower than that in 2024 (range: 13.18–21.78; *n* = 20; independent-samples *t*-test, t = 2.151, *p* = 0.038; Figure 6C). In the year with richer precipitation (2025), despite a lower bee-bread weight, the protein content was higher and the C/N ratio lower, indicating a more optimized nutritional composition.

The intertegular distance (ITD) and head width were used as indicators of the offspring’s adult body size. Morphometric results revealed that the adults emerging in 2024 were significantly smaller in body size than those in 2025. Specifically, the ITD of females in 2024 (range: 4.68–5.94 mm, *n* = 17) was significantly smaller than that of females in 2025 (range: 5.34–6.21 mm, *n* = 7; independent-samples *t*-test: t = −3.058, *p* = 0.006, Figure 6D). The same trend was observed in males: the ITD of males in 2024 (range: 4.77–5.53 mm, *n* = 23) was also significantly smaller than that of males in 2025 (range: 4.96–6.39 mm, *n* = 13; independent-samples *t*-test: t = −5.862, *p* < 0.001, Figure 6D). Similarly, the head width of females in 2024 (range: 3.72–5.07 mm, *n* = 17) was significantly smaller than that of females in 2025 (range: 4.47–4.83 mm, *n* = 7; independent-samples *t*-test: t = −2.497, *p* = 0.021, Figure 6E). The same trend was also observed in males: the head width of males in 2024 (range: 3.85–5.01 mm, *n* = 23) was significantly smaller than that of males in 2025 (range: 3.91–4.91 mm, *n* = 13; independent-samples *t*-test: t = −2.210, *p* = 0.034, Figure 6E).

## 4. Discussion

Food resources play an important role in regulating animal activities and population dynamics [49]. Large carpenter bees are generalist flower visitors, utilizing a wide range of flower shapes, sizes and colors to collect nectar [11,30,41,50]. Their generalist use of floral resources may be associated with their large body size and high energy requirements. This study observed that the *X. mongolicus* forages on 17 species in desert habitat, exhibiting generalist characteristics. However, the pollen analysis of its bee bread reveals a specialized core provisioning strategy. The pollen of the two leguminous plants, *A. mongolicus* and *O. bicolor*, together account for over 92% of the total pollen, with *A. mongolicus* making up nearly 70%. This indicates that despite its broad foraging range, the *X. mongolicus* shows a strong preference for leguminous plants, especially *A. mongolicus*, during the reproductive period, particularly when provisioning for offspring. This combination of ‘behavioral polylecty’ and ‘nutritional specialization’ in foraging and food storage has been documented in other *Xylocopa* species [11,44,51]. For instance, although *X. valga* has been recorded foraging on 95 plant species across 30 families, its bee bread in the poplar forests of Ejin Banner, China, is predominantly composed of pollen from *Sophora alopecuroides* [36,52]. This flexibility in foraging behavior serves as a key adaptive strategy for solitary bees to maintain population survival in the unpredictable resource conditions of desert habitats [39,53].

*A. mongolicus* is a unique evergreen broad-leaved shrub endemic to the desert regions of Central Asia and a major constructive species in steppified desert ecosystems. It is listed as a National Grade II Protected Wild Plant and assessed as Vulnerable. Its flowering period is concentrated in early spring (April–May), and the species exhibits traits such as cold tolerance, drought resistance, a well-developed root system, and resistance to wind and sand [54,55,56]. Wild bees are generally considered efficient pollinators of *A. mongolicus* [55,57]. This study further reveals that its peak flowering period closely overlaps with the reproductive peak of the large carpenter bee (*X. mongolicus*), thereby providing the bee with concentrated and abundant pollen and nectar resources. This tight phenological match is likely a key ecological driver behind the female bees’ specialized use of this plant during the reproductive period [51,58]. However, this study did not directly evaluate the reproductive fitness benefits that *A. mongolicus* gains through bee-mediated pollination. Future research should further elucidate the regulatory mechanisms of the mutual reproductive fitness between *X. mongolicus* and *A. mongolicus* in the context of climate change in order to better understand their mutualistic relationship in fragile ecosystems and to provide a scientific basis for relevant conservation practices.

Foraging range is an important component in the study of bee pollination ecology [23,59]. Bees are highly specialized central-place foragers whose foraging activity provides food for themselves and their offspring [32,40]. The greater the distance between the nest and food sources, the higher the energy expenditure and collection costs for bees [60,61]. Foraging success is generally determined by habitat size as well as the number and species of nectariferous plants that bees can utilize [59]. When floral resources are scarce or located far from the nest, foraging trips may take significantly longer [26]. It was previously reported that large carpenter bees are insensitive to variability in floral rewards while foraging and do not avoid resource patches with high variability in nectar rewards [34,62]. However, this study revealed that the *X. mongolicus* exhibits notable annual variation in its spatial foraging decisions. In years of low nectar resource availability (e.g., July 2023 and May 2024), female bees tended to forage conservatively on key plant species near the nesting area to minimize foraging risk. In contrast, during years of abundant resources (e.g., May and July 2025), we observed almost no female bees foraging on flowering plants near the nests. Moreover, the protein content of bee bread collected in 2025 was significantly higher, suggesting that female bees may have shifted toward long-distance “expeditionary” foraging to maximize energy gain. This ability to dynamically adjust foraging range based on resource abundance represents another key behavioral adaptation for coping with highly fluctuating conditions in desert environments, likely driven by a trade-off between energy gain and foraging cost [63].

The foraging activity of bees is jointly influenced by the climatic conditions, the nature of flowering plants, and nectar secretion levels [17]. Nectar secretion itself is directly linked to pollinator visitation and weather conditions [34]. Hot, arid environments exert varying effects on different bee species and subspecies [11,17,64]. This study demonstrates that pollen-collecting duration is significantly positively correlated with both temperature and humidity. In desert habitats, bees tend to extend their foraging bouts during more favorable periods, which may allow for more food collection or more floral visits per trip, thereby enhancing foraging efficiency. Although wind speed showed no statistically significant correlation with foraging duration, we observed in July 2023 and May 2024 at the study site that during strong wind conditions (>Force 8) on clear days, both female and male bees adopted a ground-hugging, low-altitude flight strategy upon leaving the nest and foraged predominantly on nectar plants near the nest. This indicates that they possess a flexible, immediate behavioral capacity to cope with extreme meteorological factors. Nevertheless, the underlying adaptive mechanisms to such extreme environments warrant further investigation.

The protein content of pollen is a core indicator for assessing its nutritional value [65]. For bees, the crude protein content of collected pollen typically ranges between 7.5% and 35% [66]. Protein intake is crucial for larval development [5], and increasing dietary pollen protein can significantly enhance bee survival, body size, and longevity [66,67]. Research has shown that bumblebees actively select pollen with significantly higher protein and essential amino acid content [68,69]. This study reveals that *X. mongolicus* may possess a similar adaptive capacity; its offspring provisioned with bee bread of higher protein content developed larger body sizes, confirming that food quality (rather than quantity alone) is key to determining offspring developmental success. As precipitation decreases, temperatures rise, and droughts intensify, bee species with larger body sizes, larger wings, lighter coloration, and more hair appear to be more advantaged [70]. The findings of this study further substantiate that, against the backdrop of climate fluctuation, assessing pollinator reproductive success must focus on the intrinsic nutritional quality of resources.

Drought is an increasingly severe consequence of climate change. It often leads plants to reduce investment in flower number, flower size, and nectar production [71,72,73] and, in some cases, decreases pollen quantity and viability [71]. Consequently, drought-induced changes in floral resource allocation reduce pollinator visitation [74,75,76] and plant reproductive success [77,78]. In desert ecosystems, precipitation gradients significantly influence the flowering performance of *A. mongolicus*, which is a key nectar source for the reproductive success of offspring of *X. mongolicus*. The flowering status of *A. mongolicus* is directly affected by spring rainfall [76]. Meteorological data show that winter snowfall was 12.9 mm in 2023, spring rainfall was 7.8 mm in 2024, winter snowfall was 2.9 mm in 2024, and spring rainfall was 15.7 mm in 2025. This study found that flowering status in 2025 was significantly better than that in 2024 (Figure 1C,D), which may be attributed to the relatively abundant spring rainfall in 2025. The results also revealed that in 2025, reproductive females stored less bee bread in brood cells, but the protein content was higher, ultimately resulting in larger offspring body size. These findings further suggest that in desert ecosystems, the alignment of precipitation timing with phenological events is more critical than total precipitation amount, with spring rainfall potentially serving as an important ecological factor influencing wild bee reproduction.

## 5. Conclusions

In summary, the *X. mongolicus* adopts a composite strategy of “elastic foraging, quality focusing” to adapt to harsh desert environments. Its reproductive success is highly dependent on the pollen quality of key nectar plants (e.g., *A. mongolicus*), which is directly regulated by precipitation conditions before and during the flowering period. Consequently, under climate change, shifts in precipitation patterns may profoundly affect the population recruitment and long-term persistence of this bee species by influencing the physiological status and nutritional output of key plants. This study highlights that conserving key pollinators in desert ecosystems requires not only protecting their habitats and plant diversity but also understanding how climate fluctuations alter the nutritional foundation of plant–pollinator interactions, ultimately shaping population health.

## Figures and Tables

**Table 1:** Characteristics of plant species utilized as food resources by *X. mongolicus.*

Family	Plant Species	Plant Type	Flowering Period	Flower Size	Flower Type	Floral Symmetry	Floral Color	Floral Reward
Fabaceae	*Ammopiptanthus mongolicus*	Shrub	Apr.–May	Medium	Papilionaceous corolla	Zygomorphic	Yellow	Pollen and nectar
	*Oxytropis bicolor*	Herb	May–June	Small	Papilionaceous corolla	Zygomorphic	Purplish red, bluish purple	Pollen and nectar
	*Astragalus melilotoides*	Herb	July–Aug.	Small	Papilionaceous corolla	Zygomorphic	White or pink	Pollen and nectar
Asteraceae	*Lipschitzia divaricata*	Herb	June–Aug.	Medium	Ligulate corolla	Zygomorphic	Yellow	Pollen
	*Echinops gmelinii*	Herb	June	Small	Tubular corolla	Actinomorphic	Blue	Pollen and nectar
	*Olgaea leucopluylla*	Herb	June–Sep.	Small	Tubular corolla	Actinomorphic	Purple or white	Pollen and nectar
Brassicaceae	*Dontostemon dentatus*	Herb	May–July	Medium	Cruciform corolla	Actinomorphic	light purple	Pollen and nectar
Papaveraceae	*Hypecoum erectum*	Herb	May–Aug.	Medium	—	Actinomorphic	pale yellow	Pollen
Nitrariaceae	*Peganum harmala*	Herb	May–June	Medium	—	Actinomorphic	Yellow white	Pollen and nectar
Boraginaceae	*Tournefortia sibirica*	Herb	May–June	Small	Funnelform corolla	Actinomorphic	White	Pollen and nectar
Convolvulaceae	*Convolvulus ammannii*	Herb	July–Sep.	Medium	Funnelform corolla	Actinomorphic	Light pink or white with purple stripes	Pollen
	*Convolvulus arvensis*	Herb	June–Aug.	Medium	Funnelform corolla	Actinomorphic	pink	Pollen and nectar
Rosaceae	*Potentilla supina*	Herb	May–Sep.	Small	Rosaceous corolla	Actinomorphic	Yellow	Pollen and nectar
	*Rosa xanthina*	Shrub	May–June	Large	Rosaceous corolla	Actinomorphic	Yellow	Pollen and nectar
	*Rosa rugosa*	Shrub	June–Aug.	Large	Rosaceous corolla	Actinomorphic	Purplish red	Pollen and nectar
Iridaceae	*Iris lactea*	Herb	May–June	Large	—	Actinomorphic	Bluish purple	Pollen and nectar
Plantaginaceae	*Plantago minuta*	Herb	June–Aug.	Small	—	Actinomorphic	White	Pollen and nectar

## Data Availability

The data are not publicly available due to privacy or ethical restrictions.
